# Integrating Machine Learning to Identify Key Microbiota of Gut Community Changes Across Different Stages in Dahe Black Pigs

**DOI:** 10.3390/microorganisms13092038

**Published:** 2025-08-31

**Authors:** Lanlan Yi, Wenjie Cheng, Guangyao Song, Huijin Jia, Yuxiao Xie, Wanghong Zhang, Junhong Zhu, Sumei Zhao

**Affiliations:** College of Animal Science and Technology, Yunnan Agricultural University, Kunming 650201, China; yilanlan0217@163.com (L.Y.); cwj210365@163.com (W.C.); songguangyao1990@163.com (G.S.); 2022210413@stu.ynau.edu.cn (H.J.); xieyuxiao@zync.edu.cn (Y.X.); zwhzdk@126.com (W.Z.)

**Keywords:** 16S rRNA sequencing, machine learning, *Escherichia shigella*, *Lactobacillus*, *Lachnospiraceae XPB1014 group*, *Clostridium sensu stricto 1*

## Abstract

Growth stage is a key factor influencing the composition and richness of the porcine gut microbiota. The stage-specific alterations in gut microbiota of indigenous Chinese pig breeds and cultivated breeds remain to be elucidated. This study conducted 16S rRNA sequencing analysis on fecal microbiota from Dahe black pigs across distinct growth stages. Samples included lactating sows, suckling piglets, weaned piglets, pigs weighing 50–100 kg, pigs weighing 120–150 kg, and pigs weighing > 200 kg. The results indicated that *Escherichia shigella* (12.4% vs. 16.2%), *Lactobacillus* (5.9% vs. 6.3%), and *Rikenellaceae RC9 gut group* (3.9% vs. 4.2%) were dominant genera shared between lactating sows and suckling piglets. The relative abundance of *Eubacterium brachy group* was significantly higher in lactating sows, whereas *Flavonifractor* was significantly lower compared to suckling piglets (*p* < 0.05). Compared to pigs weighing > 120 kg, lactating sows exhibited 22 differentially abundant genera, including *Escherichia shigella*, *Cloacibacillus*, *Fusobacterium*, *Faecalibacterium*, and *Prevotella* (*p* < 0.05). In suckling piglets, Firmicutes and Bacteroidota constituted 47.4% and 27.3% of the microbiota, respectively. Their relative abundance increased with body weight, reaching 52.6% and 33.3% in pigs weighing > 200 kg. Proteobacteria decreased from 17.3% in suckling piglets to 2.0% in >200 kg pigs. Spirochaetota declined from 2.5% in suckling piglets to 0.9% in weaned piglets and then increased to 6.9% in >200 kg pigs. *Lactobacillus* peaked at 15.7% in weaned piglets, while *Escherichia shigella* reached its maximum (16.2%) in suckling piglets, both gradually declining thereafter. *Streptococcus* abundance remained relatively stable (1.1% in suckling piglets; 4.5% in weaned piglets). *Prevotellaceae NK3B31 group* registered 2.9% in suckling piglets, increased to 7.1% in weaned piglets, and then declined to 2.6% in >200 kg pigs. *Mitsuokella*, *Bilophila*, *Succinivibrio*, *Romboutsia*, and *Desulfovibrio* were identified as the top five genera discriminating suckling and weaned piglets. Similarly, *Lachnospiraceae XPB1014 group*, *Clostridium sensu stricto 1*, *Turicibacter*, *Quinella*, and *p 1088 a5 gut group* were key discriminators between weaned piglets and 50–100 kg pigs. These identified microbial taxa represent potential candidate targets for modulating the developmental timing of growth phases in pigs, offering possibilities for either advancing or delaying specific physiological timepoints.

## 1. Introduction

The composition of the gut microbiota varies substantially across individuals and developmental stages, with the bacterial domain constituting the predominant component in swine [1]. Early gut colonizers in pigs are critical for establishing a permanent microbial community architecture that exerts enduring influences on porcine health and growth performance [2]. Unlike the relatively stable gut microbiota in adults, the neonatal microbiota dramatically fluctuates, exhibiting high adaptability and plasticity while being continually influenced by maternal factors through direct skin contact and breastfeeding after birth [3]. A meta-analysis of 3313 fecal samples across 60 timepoints (0–183 days) spanning six countries and four continents identified “study” and growth stage as the primary drivers of swine gut microbiota variation, outweighing age, hypervariable region, sequencing platform, and country of origin [4]. Host age was the dominant factor in shaping the gut microbiota of piglets after weaning, which follows a highly structured developmental program characterized by post-weaning changes [5,6]. Gut microbial diversity undergoes continuous age-dependent shifts, with aged pigs typically exhibiting reduced beneficial taxa and increased abundance of Bacteroides [7].

During porcine development, microbial succession drives dynamic compositional changes in the gut microbiome, which exhibits clear spatial variation along the gastrointestinal tract and is dominated by Firmicutes and Bacteroidetes [8]. Dominating the porcine gut from birth to 2 days, Escherichia, Clostridium, Fusobacterium, Streptococcus, and Enterococcus genera establish initial colonization, while Clostridiaceae (predominant at 6 h postnatal) decline to ≤1% abundance by day 20, and Enterobacteriaceae surge by post-weaning day 5 before significantly reducing after post-weaning day 11 [9]. Compositional shifts across porcine growth stages (suckling, weaning, nursery, grower, finisher) reveal 19 core genera (dominated by Bacteroides, Prevotella, and Lactobacillus) with both these “core” bacteria and enterotypes invariably transitioning to an adult-like profile with age [10]. Distinct developmental stages in pigs exhibit stage-specific gut microbial profiles.

Through prolonged artificial selection under diverse natural environments, Chinese indigenous pig breeds have undergone significant alterations in the relative abundance of their gut microbiota. In Wuzhishan pigs, a Chinese indigenous miniature breed, the abundance of Firmicutes and fecal microbial diversity increased progressively across four developmental stages: pre-weaning piglet, weaning piglet, growing pig, and sow, while the proportions of Bacteroidetes declined [11]. Fresh fecal samples from six Tunchang pig stages (pre-weaning/weaning piglets, growing pigs, suckling/empty sows, adult males) showed increased Firmicutes and Spirochaetes with decreased Bacteroidetes and Proteobacteria over development [12]. However, among China’s 83 indigenous pig breeds and 38 cultivated breeds, the gut microbial developmental characteristics of numerous porcine genetic resources remain systematically uncharacterized.

Machine learning has gained increasing prevalence across diverse fields, including microbiome data analysis. Distinct microbial genera were employed as predictors for the prevalence of foodborne pathogens during key broiler growth phases: the START phase (weeks 2–4), MID phase (weeks 5–7), and END phase (weeks 8–11) [13]. Machine learning integrated with Shapley Additive Explanation (SHAP) methodology delivers interpretable insights into models that identify signature microbiota based on milk urea nitrogen concentrations [14]. Current research on the developmental stages of the gut microbiota in Dahe black pigs remains limited. Machine learning offers a viable approach to identifying pivotal microbial taxa characteristic of distinct growth phases, thereby laying the groundwork for subsequent strain selection and validation. The present study selected Dahe black pigs—a cultivated breed derived from Dahe pigs and Duroc genetics—as the experimental model. We comprehensively profiled the intestinal microbial features using 16S rRNA sequencing across distinct developmental stages in this breed. By integrating machine learning approaches, stage-specific core microorganisms were identified. This study identifies potential microbial candidates for screening that may temporally modulate developmental stages through subsequent probiotic supplementation.

## 2. Materials and Methods

### 2.1. Animals and Sample Collection

All the animal procedures were approved by the Animal Care and Use Committee of Yunnan Agricultural University (approval code 202403081). Fecal samples were collected from pigs at an ecological farm in Fuyuan County, Qujing City, Yunnan Province, China. Environmental variables were strictly controlled throughout the study. All 323 fecal samples were collected within a single month (April 2024) to eliminate seasonal effects. Sows were individually housed and received feed devoid of probiotics, antibiotics, or veterinary medications. Piglets followed standardized developmental protocols: creep feeding initiated at postnatal day 14, weaning at day 28 with immediate transfer to nursery pens, and entry into the fattening phase at day 70. Consistent hygiene practices, feeding regimes, and environmental conditions were maintained within each production phase according to the farm’s standard operating procedures (SOPs). These samples included lactating sows (SE, n = 10), lactating piglets (SP, n = 55), weaned piglets (WP, n = 54), pigs weighing 50–100 kg (P50_100, n = 52), pigs weighing 120–150 kg (P120_150, n = 101), and pigs weighing more than 200 kg (P200, n = 51). Only the internal portion of the feces was collected. All animals received stage-specific antibiotic-free diets meeting or exceeding nutritional requirements recommended by NRC (2012).

### 2.2. Microbial DNA Extraction and Sequencing

DNA was extracted from fecal samples using the HiPure Stool DNA Kit (Magen, Guangzhou, China) according to the manufacturer’s protocol. The V3-V4 region of bacterial 16S rRNA genes was amplified with primers 341F (5′-CCTACGGGNGGCWGCAG-3′) and 806R (5′-GGACTACHVGGGTATCTAAT-3′). PCR products were purified with AMPure XP Beads (Beckman Coulter, Brea, CA, USA), quantified using a Qubit 3.0 Fluorometer, and constructed into sequencing libraries with the Illumina DNA Prep Kit (Illumina, San Diego, CA, USA). Library quality was assessed on an ABI StepOnePlus Real-Time PCR System (Life Technologies, Foster City, CA, USA). Qualified libraries were sequenced on the Illumina NovaSeq 6000 platform with PE250 configuration.

### 2.3. Sequencing Data Analysis

Sequencing data were processed using FASTP (v0.18.0) to remove reads containing (1) ≥10% unidentified nucleotides (N), (2) >50% bases with Phred quality scores ≤20, or (3) adapter sequences [15]. Clean reads were assembled into tags via FLASH (v1.2.11) with a minimum overlap of 10 bp and a maximum mismatch rate of 2% [16]. Raw tags were truncated at the first base position where consecutive low-quality bases (Q ≤ 3) reached a length threshold of 3. Post-truncation tags were further filtered by discarding those with consecutive high-quality base lengths < 75% of total tag length [17]. Clean tags were clustered into operational taxonomic units (OTUs) at a 97% similarity threshold using the UPARSE algorithm in USEARCH (v11.0.667) [18]. Chimeric sequences were detected and removed via UCHIME, with subsequent analyses performed on effective tags [19].

OTU sequences were taxonomically annotated against the SILVA database (v138.2) [20]. Species composition was visualized using stacked bar plots generated with the R package ggplot2 (v2.2.1) [21]. Venn diagrams were constructed with the R package VennDiagram [22]. Differential microbial features were identified via LEfSe using thresholds of LDA score ≥ 2.0 and *p* < 0.05 [23]. Alpha diversity indices (Shannon/Simpson) and beta diversity (PCoA based on Bray–Curtis distance) were computed using the vegan package in R for both pairwise and multi-group comparisons [24].

### 2.4. Machine Learning Based on the Fecal Microbiome

The K-medoids algorithm (also termed PAM, Partitioning Around Medoids) is a standard clustering method for microbial community typing, grouping samples with analogous species composition into distinct clusters. For each cluster, a medoid is computed. Through iterative optimization, the algorithm minimizes the total sum of dissimilarity distances between all samples. The optimal K value (number of clusters) is determined by maximizing the average silhouette width, which quantitatively evaluates cluster cohesion and separation.

The SHAP algorithm treats each feature as a “contributor” to the prediction outcome, explaining its importance in specific predictions made by ML and random forest models. SHAP quantifies each feature’s contribution to the model output by assigning a Shapley value, which indicates both the direction and magnitude of the feature’s influence [25]. For a specific prediction, the cumulative Shapley values across all features plus the average prediction value yield the model’s final output, thereby revealing individual feature contributions. Feature contributions derived from 16S rRNA data are determined by the sum of the cumulative SHAP values for a specific prediction and the average prediction value. Global interpretation computes the mean SHAP value for each feature across all samples, highlighting overall feature importance, whereas local interpretation assesses the impact of each feature on an individual prediction [26].

### 2.5. Statistical Analysis

Machine learning analysis was performed using Python (version 3.10.2). Prior to model construction, the dataset was randomly split into training (70%) and testing (30%) sets. Significance analyses were conducted using R software (version 4.1.1) and SPSS 22.0. The Mann–Whitney U test was employed for two-group comparisons, while the Kruskal–Wallis test was applied for multi-group comparisons. A threshold of *p* < 0.05 was considered statistically significant. Permutational multivariate analysis of variance (PERMANOVA) was performed using the adonis function (vegan package) to assess significant differences between groups. The graphical elements in the abstract were sourced from BioRender (https://www.biorender.com/) and created using draw.io software (v22.0.2).

## 3. Results

### 3.1. Vertical Transmission of Gut Microbiota from Sows to Offspring

**Figure 1:**
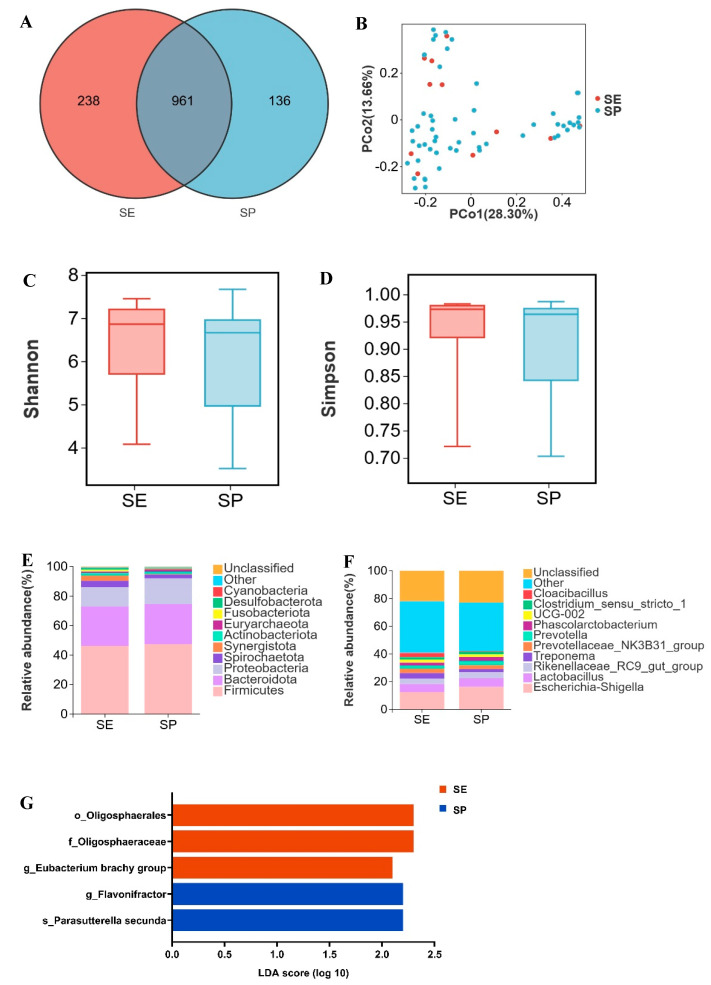
Vertical transmission of microbiota from Dahe sows to their offspring. (**A**) Venn diagram of OTUs between lactating sows (SE) and suckling piglets (SP), (**B**) two-dimensional PCoA plot of all SE and SP samples, (**C**) Shannon index comparison (Mann–Whitney U test, *p* = 0.3), (**D**) Simpson index comparison (Mann−Whitney U test, *p* = 0.4), (**E**) top 10 phyla by relative abundance in SE and SP, (**F**) top 10 genera by relative abundance in SE and SP (unclassified = unannotated microorganisms, other = unlisted taxa), (**G**) LEfSe analysis (“o_” = order, “f_” = family, “g_” = genus, “s_” = species).

SE and SP shared 961 OTUs, with 238 OTUs unique to SE and 136 OTUs unique to SP (Figure 1A). Principal coordinates analysis (PCoA) based on Bray–Curtis distance revealed distinct clustering of SE and SP samples (Figure 1B). No significant differences were observed in Shannon or Simpson indices (*p* > 0.05; Figure 1C,D). Both SE and SP exhibited dominant phyla Firmicutes (46.3% vs. 47.4%), Bacteroidota (26.7% vs. 27.3%), and Proteobacteria (13.1% vs. 17.3%) (Figure 1E). The relative abundance of Synergistota was higher in SE (3.6%) than in SP (0.5%). Dominant genera shared by SE and SP included *Escherichia shigella* (12.4%, 16.2%), *Lactobacillus* (5.9%, 6.3%), and *Rikenellaceae RC9 gut group* (3.9%, 4.2%) (Figure 1F). *Cloacibacillus* (in phyla Synergistota) demonstrated greater abundance in SE versus SP. Using LDA > 2 and *p* < 0.05 as thresholds, differential microbial analysis revealed significantly elevated abundance of *Eubacterium brachy group* in SE compared to SP, whereas *Flavonifractor* showed reduced abundance in SE (Figure 1G, Table S1). Overall, the microbiota composition of the SE and SP groups was similar, with observed differences mainly due to changes in the relative abundance of specific bacterial genera.

### 3.2. Differential Gut Microbial Abundance Between Lactating Sows and Adult Swine

**Figure 2:**
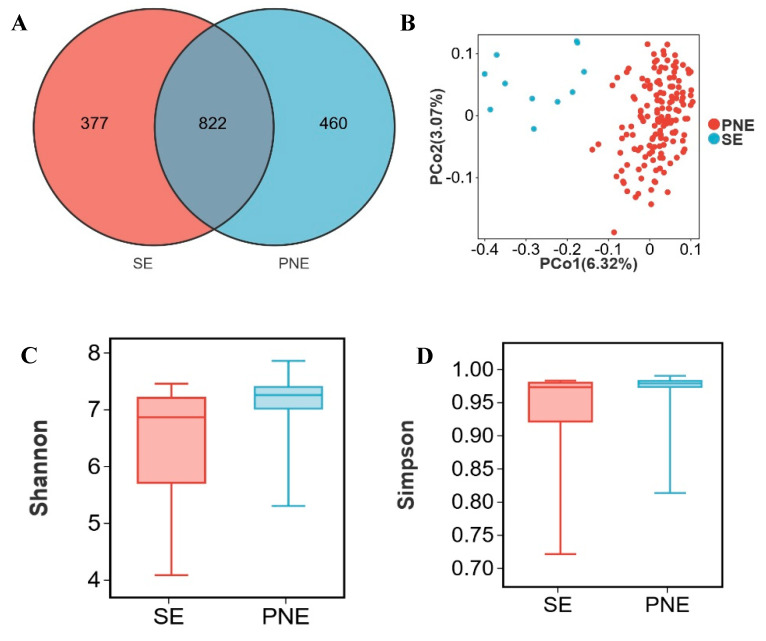

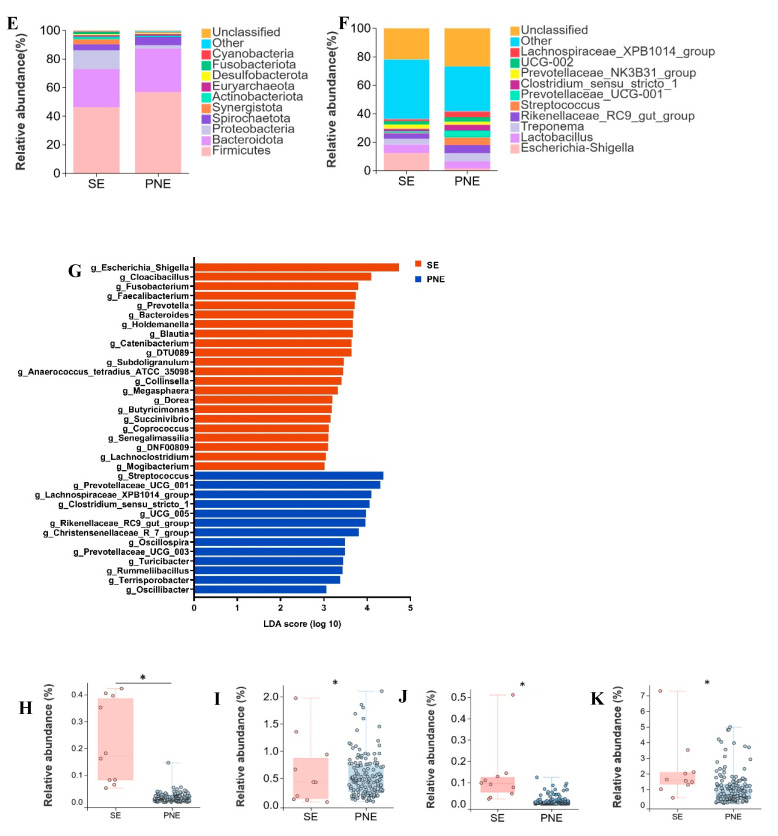
Distinct characteristic of microbial relative abundance during the lactation period. (**A**) Venn diagram of OTUs between lactating sows (SE) and pigs weighing > 120 kg (PNE), (**B**) two- dimensional PCoA plot of all SE and PNE samples, (**C**) Shannon index comparison (Mann–Whitney U test, *p* = 0.01), (**D**) Simpson index comparison (Mann–Whitney U test, *p* = 0.04), (**E**) top 10 phyla by relative abundance in SE and PNE, (**F**) top 10 genera by relative abundance in SE and PNE, (**G**) analysis of bacterial genera with LDA scores greater than 3 and *p* < 0.05 using LEfSe, “g_” = genus level, (**H**) the relative abundance of *Lachnoclostridium*, (**I**) the relative abundance of *Ruminococcus*, (**J**) the relative abundance of *Ruminococcus torques group*, (**K**) the relative abundance of *Prevotella*. * *p* < 0.05.

Piglet gut microbiota primarily derives from sows, with significant compositional differences observed between lactating sows (SE; body weight: 150–200 kg) and market-weight pigs. Integration of gut microbiota from all pigs ≥ 120 kg (designated PNE) enabled analysis of microbial alterations in lactating sows. Although 822 OTUs were shared between SE and PNE, 377 OTUs were unique to SE and 460 to PNE (Figure 2A). Principal coordinates analysis demonstrated distinct clustering patterns separating SE and PNE (Figure 2B). A significant difference was indeed observed between the SE and PNE groups (PERMANOVA, *p* = 0.001, R^2^ = 0.0878). The Shannon and Simpson indices were significantly lower in SE compared to PNE (*p* < 0.05) (Figure 2C,D).

Firmicutes (56.8%, 46.3%), Bacteroidota (30.6%, 26.7%), Proteobacteria (2.2%, 13.1%), and Spirochaetota (5.9%, 4.2%) were predominant in the feces of PNE and SE (Figure 2E). The relative abundance of *Escherichia shigella* in SE (12.4%) was higher than in PNE (1.4%). In contrast, the levels of *Streptococcus*, *Prevotellaceae UCG 001*, and *Clostridium sensu stricto 1* were lower in SE (0.5%, 0.9%, and 1.7%, respectively) compared to PNE (5.3%, 4.9%, and 4.0%) (Figure 2F). Using LDA > 2.0 and *p* < 0.05 as thresholds to screen for differential microbes, 59 differential genera were identified in the SE group and 32 differential genera in the PNE group (Supplementary Table S2). Genera with LDA scores > 3.0 were filtered from Supplementary Table S2. The SE group exhibited 22 differential genera, including *Escherichia shigella*, *Cloacibacillus*, *Fusobacterium*, *Faecalibacterium*, and *Prevotella*, whereas the PNE group contained 13 differential genera, such as *Streptococcus*, *Prevotellaceae UCG 001*, *Lachnospiraceae XPB1014 group*, *Clostridium sensu stricto 1*, and *UCG-005* (Figure 2G). The relative abundances of *Lachnoclostridium*, *Ruminococcus*, *Ruminococcus torques group*, and *Prevotella* in lactating sow fecal samples were higher than in other pigs over 120 kg.

### 3.3. Changes of Gut Microbiota Across Porcine Growth Stages

**Figure 3:**
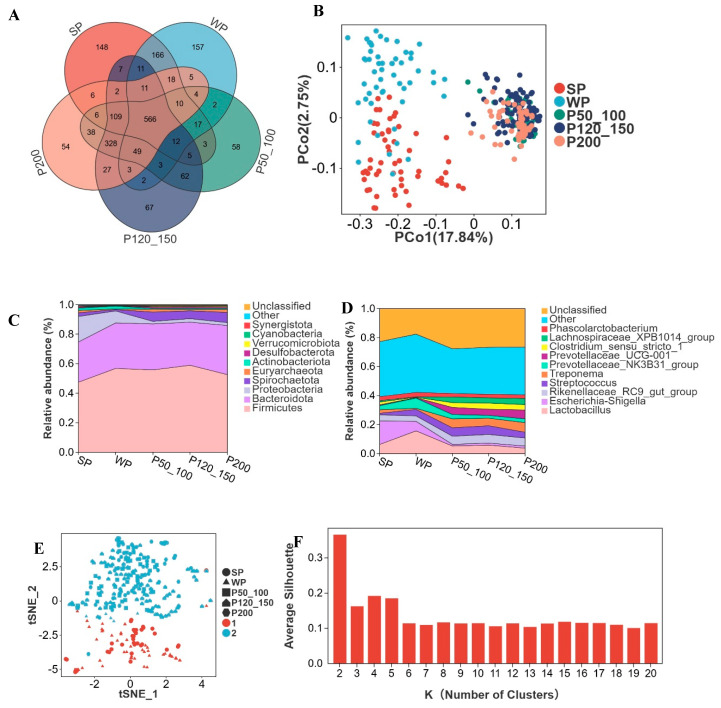
Advancing age accompanies alterations in microbial composition and relative abundance. (**A**) Venn diagram of OTUs across developmental stages, suckling piglets (SP), weaned piglets (WP), pigs weighing 50–100 kg (P50_100), 120–150 kg (P120_150), and >200 kg (P200), (**B**) two-dimensional PCoA plot of all samples, (**C**) top 10 phyla by relative abundance, (**D**) top 10 genera by relative abundance, (**E**) cluster-based regrouping of samples via PCoA, (**F**) cluster count determination with silhouette width metric, where higher values indicate superior clustering.

In different growth stages of Dahe black pigs (including SP, WP, P50_100, P120_150, and P200), 566 OTUs were shared among all groups, while 148, 157, 58, 67, and 54 OTUs were uniquely identified in each respective stage (Figure 3A). During the SP and WP stages, the gut microbiota of Dahe black pigs underwent substantial changes, with significant variations in microbial composition and relative abundance among individuals. However, microbial profiles gradually converged with age, exhibiting increased similarity (Figure 3B).

Firmicutes and Bacteroidota constituted 47.4% and 27.3%, respectively, at the SP stage, with their relative abundance increasing as body weight grew, reaching 52.6% and 33.3% at P200 (Figure 3C). Proteobacteria accounted for 17.3% at the SP stage but progressively decreased to 2.0% by P200. Spirochaetota represented 2.5% at SP, declined to 0.9% at WP, and then subsequently increased to 6.9% at P200. *Lactobacillus* peaked at 15.7% in WP, while *Escherichia shigella* reached its highest level at 16.2% in SP, both gradually decreasing thereafter (Figure 3D). The relative abundance of *Streptococcus* was 1.1% in SP and 4.5% in WP, remaining relatively stable thereafter. *Prevotellaceae NK3B31 group* registered 2.9% in SP, increased to 7.1% in WP, and then declined to 2.6% at P200. *Treponema*, *Prevotellaceae UCG 001*, *Clostridium sensu stricto 1*, and *Lachnospiraceae XPB1014 group* maintained relative abundances below 3% during SP/WP but exceeded 3% in P50_100, P120_150, and P200 stages. The K-medoids algorithm partitioned all genera into two clusters. SP and WP were grouped into Cluster 1, while P50_100, P120_150, and P200 formed Cluster 2 (Figure 3E,F).

### 3.4. Critical Transition of Microbial Abundance from Suckling to Weaned Piglets

**Figure 4:**
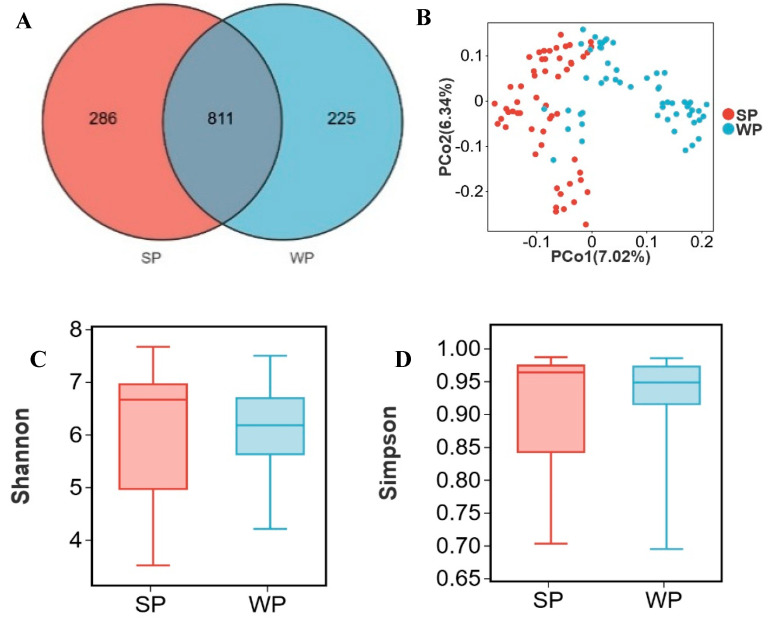

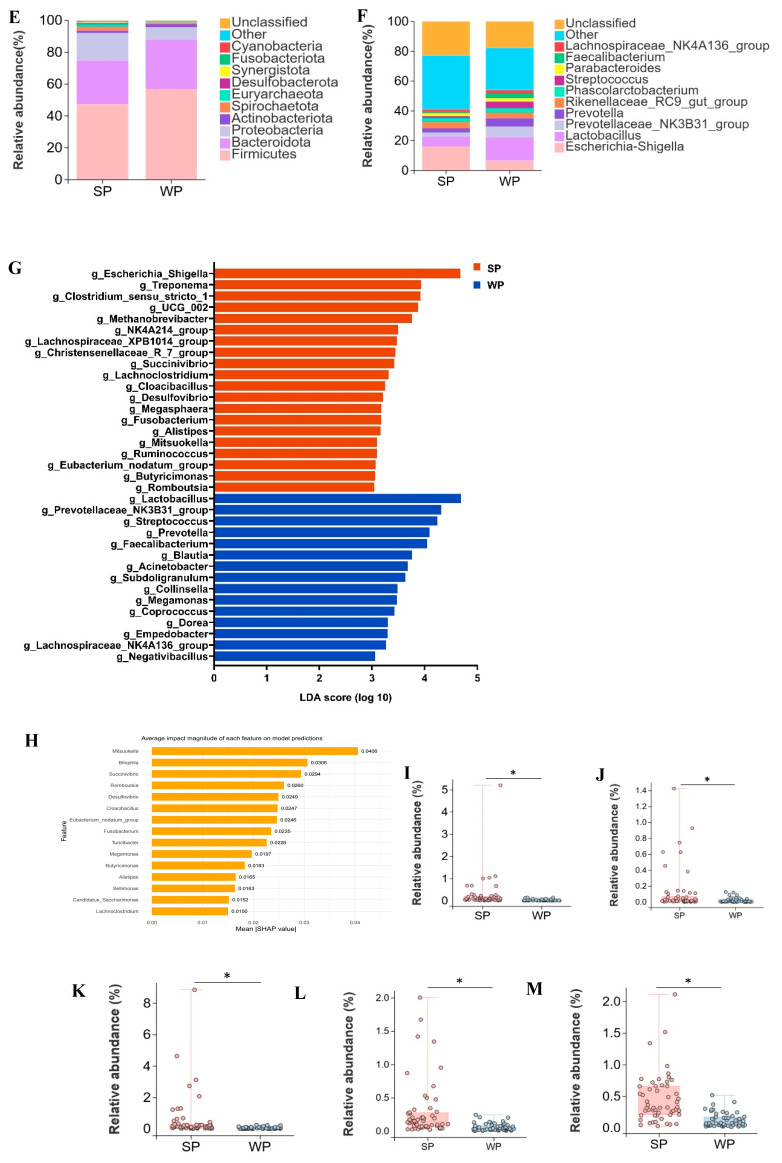
Alterations in gut microbiota from suckling to weaned piglets. (**A**) Venn diagram of OTUs between suckling piglets (SP) and weaned piglets (WP), (**B**) two-dimensional PCoA plot of all SP and WP samples, (**C**) Shannon index comparison (Mann–Whitney U test, *p* = 0.4), (**D**) Simpson index comparison (Mann–Whitney U test, *p* = 0.7), (**E**) top 10 phyla by relative abundance in SP and WP, (**F**) top 10 genera by relative abundance in SP and WP, (**G**) analysis of bacterial genera with LDA scores greater than 3 and *p* < 0.05 using LEfSe, (**H**) SHAP interprets and visualizes machine learning outcomes, (**I**–**M**) the relative abundance of *Mitsuokella* (**I**), *Bilophila* (**J**), *Succinivibrio* (**K**), *Romboutsia* (**L**), and *Desulfovibrio* (**M**). * *p* < 0.05.

SP and WP shared 811 OTUs, while SP harbored 286 unique OTUs and WP contained 225 distinct OTUs (Figure 4A). Approximately two-thirds of individual samples formed separate clusters in PCoA ordination between SP and WP groups (Figure 4B). No significant differences (*p* > 0.05) were observed in the Shannon and Simpson indices between the two groups (Figure 4 C, D). At the phylum level, the dominant bacterial phyla in both SP and WP groups were Firmicutes (SP: 47.4%, WP: 56.8%), Bacteroidota (SP: 27.3%, WP: 30.9%), Proteobacteria (SP: 17.3%, WP: 8.1%), Actinobacteriota (SP: 1.6%, WP: 1.9%), and Spirochaetota (SP: 2.5%, WP: 0.9%) (Figure 4E). At the genus level, the relative abundances of *Escherichia shigella* (16.2%, 6.6%), *Lactobacillus* (6.3%, 15.7%), *Prevotellaceae NK3B31 group* (2.9%, 7.1%), *Prevotella* (3.0%, 5.7%), *Streptococcus* (1.1%, 4.5%), *Parabacteroides* (2.0%, 2.3%), *Faecalibacterium* (0.9%, 3.2%), and *Lachnospiraceae NK4A136 group* (1.7%, 2.1%) changed between the WP and SP groups (Figure 4F). Using an LDA score > 2 and *p* < 0.05 as thresholds, 63 differentially abundant genera were identified in the SP group and 46 in the WP group (Supplementary Table S3). Genera with LDA scores >3.0 were filtered from Supplementary Table S3. The SP group comprised 20 differential genera, including *Escherichia shigella*, *Treponema*, *Clostridium sensu stricto 1*, *UCG-002*, and *Methanobrevibacter*, while the WP group encompassed 15 differential genera, such as *Lactobacillus*, *Prevotellaceae NK3B31 group*, *Streptococcus*, *Prevotella*, and *Faecalibacterium* (Figure 4G). *Mitsuokella*, *Bilophila*, *Succinivibrio*, *Romboutsia*, and *Desulfovibrio* were identified as the top five genera contributing most significantly to distinguishing between the SP and WP groups (Figure 4H, Supplementary Table S4). The relative abundance of these genera was significantly higher in SP compared to WP (*p* < 0.05) (Figure 4I–M).

### 3.5. Changes in Microbial Abundance from Post-Weaning to Finishing Stage (50–100 kg Body Weight)

**Figure 5:**
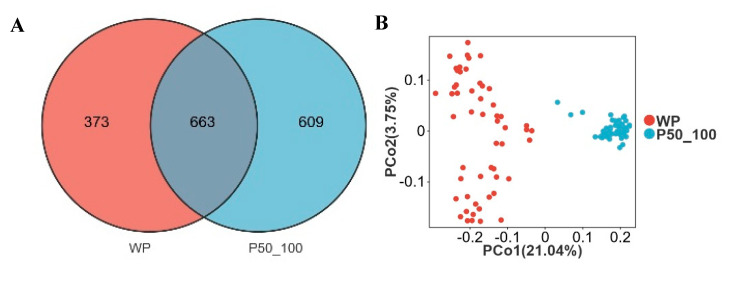

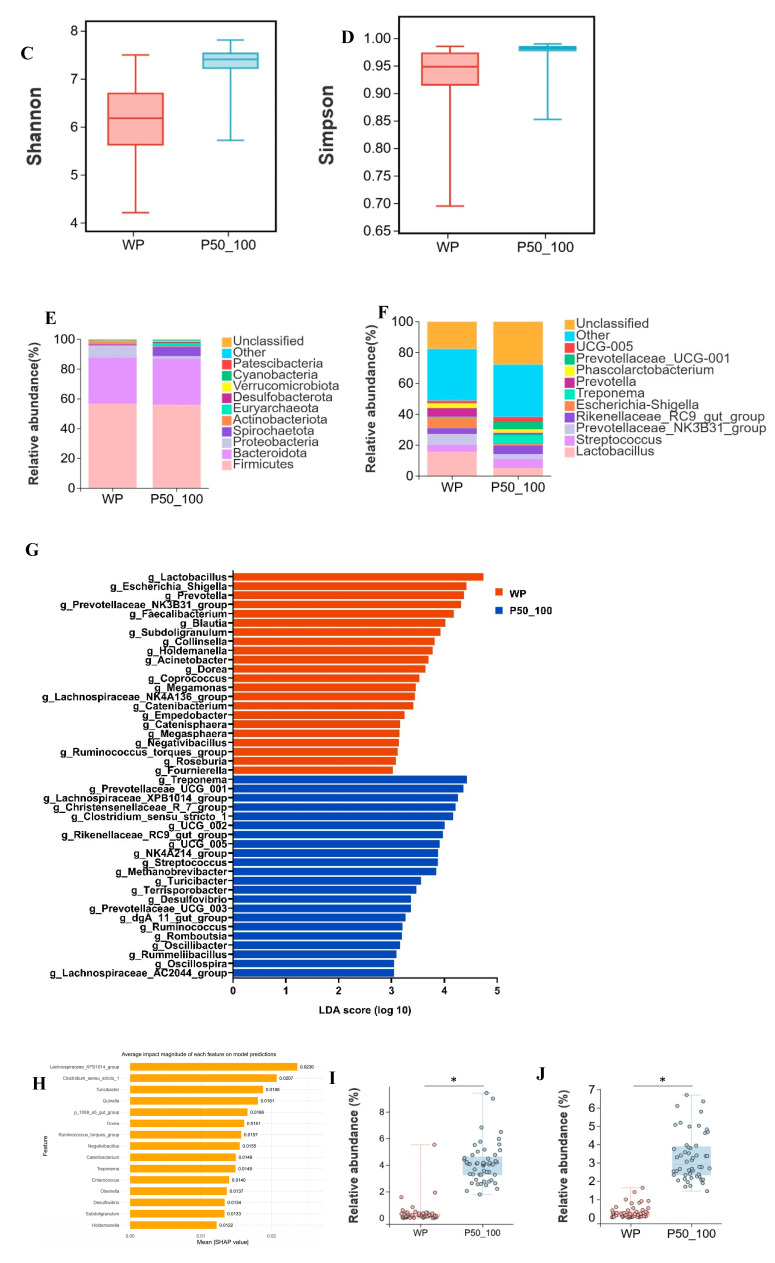

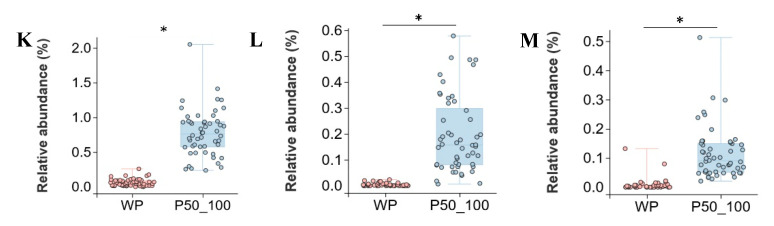
The period from weaned piglets to the 50–100 kg growth stage constitutes a critical transition point for microbial alterations. (**A**) Venn diagram of OTUs between weaned piglets (WP) and pigs weighing 50–100 kg (P50_100), (**B**) two-dimensional PCoA plot of all WP and P50_100 samples, (**C**) Shannon index comparison (Mann–Whitney U test, *p* < 0.01), (**D**) Simpson index comparison (Mann–Whitney U test, *p* < 0.01), (**E**) top 10 phyla by relative abundance, (**F**) top 10 genera by relative abundance, (**G**) analysis of bacterial genera with LDA scores greater than 3 and *p* < 0.05 using LEfSe, (**H**) SHAP interprets and visualizes machine learning outcomes, (**I**–**M**) the relative abundance of *Lachnospiraceae XPB1014 group* (**I**), *Clostridium sensu stricto 1* (**J**), *Turicibacter* (**K**), *Quinella* (**L**), and *p 1088 a5 gut group* (**M**). * *p* < 0.05.

The WP and P50_100 stages represent the pivotal transition phase where intestinal microbiota succession in Dahe black pigs culminates in stabilization. WP and P50_100 shared 663 OTUs, while WP and P50_100 harbored 373 and 609 unique OTUs, respectively (Figure 5A). PCoA revealed distinct clustering of WP and P50_100 groups, with microbial composition converging as age increased (Figure 5B). Both the Shannon and Simpson indices were significantly higher in the P50_100 group compared to WP (Figure 5C,D). Firmicutes (WP: 56.8%, P50_100: 55.7%) and Bacteroidota (WP: 30.9%, P50_100: 31.2%) dominated the fecal microbiota in both groups (Figure 5E). The relative abundance of Proteobacteria was higher in WP (8.1%) than in P50_100 (1.7%), whereas Spirochaetota abundance was lower in WP (0.9%) versus P50_100 (6.3%). Distinct genus-level community composition was observed between groups (Figure 5F). Specifically, *Lactobacillus* (15.7% vs. 5.2%), *Prevotellaceae NK3B31 group* (7.1% vs. 3.1%), *Escherichia shigella* (6.6% vs. 1.2%), and *Prevotella* (5.7% vs. 0.9%) were enriched in WP relative to P50_100. Conversely, *Rikenellaceae RC9 gut group* (3.8% vs. 5.6%), *Treponema* (0.7% vs. 6.1%), *Prevotellaceae UCG 001* (0.2% vs. 4.9%), and *UCG-005* (1.5% vs. 3.1%) showed reduced abundance in WP. LEfSe analysis identified 67 and 61 differentially abundant genera in the WP and P50_100 groups, respectively (Supplementary Table S5). Genera with LDA scores > 3.0 were filtered from Supplementary Table S4. The WP group comprised 22 differential genera, including *Lactobacillus*, *Escherichia shigella*, *Prevotella*, *Prevotellaceae NK3B31 group*, and *Faecalibacterium*, while the P50_100 group encompassed 22 differential genera, such as *Treponema*, *Prevotellaceae UCG 001*, *Lachnospiraceae XPB1014 group*, *Christensenellaceae R7 group*, and *Clostridium sensu stricto 1* (Figure 5G). The machine learning model selected *Lachnospiraceae XPB1014 group*, *Clostridium sensu stricto 1*, *Turicibacter*, *Quinella*, and *p 1088 a5 gut group* as the top five contributing microbes discriminating WP and P50_100 (Figure 5H, Supplementary Table S6). Consistently, the relative abundance of *Lachnospiraceae XPB1014 group*, *Clostridium sensu stricto 1*, *Turicibacter*, *Quinella*, and *p 1088 a5 gut group* were significantly elevated in P50_100 compared to WP (*p* < 0.05) (Figure 5I–M).

## 4. Discussion

Gut microbiota influences vital body processes, with early-life colonization being crucial for shaping intestinal development [27]. In this study, the fecal microbiota of 323 Dahe black pigs across different growth stages were analyzed to characterize their composition and abundance features using 16S rRNA sequencing. The results indicate that early-life microbial colonization in Dahe black piglets relies heavily on the sow’s microbiota. Weaning represents a critical turning point, after which microbial communities gradually stabilize in composition and richness.

Both Dahe black sows and piglets exhibited dominant phyla Firmicutes (46.3% vs. 47.4%), Bacteroidota (26.7% vs. 27.3%), and Proteobacteria (13.1% vs. 17.3%); the dominant genera included *Escherichia shigella* (12.4% vs. 16.2%), *Lactobacillus* (5.9% vs. 6.3%), and *Rikenellaceae RC9 gut group* (3.9% vs. 4.2%). The fecal microbiota of newborn piglets initially resembled environmental sources (slatted floor, sow’s milk, nipple surfaces) but proved transient, ultimately being succeeded by sow fecal microbiota [28]. Sow milk microbiota shapes piglet gut microbiomes, with *Corynebacterium* and *Streptococcus* being dominant in colostrum, while *Lactobacillus*, Ruminococcaceae, Lachnospiraceae, and Clostridiales are enriched in mature milk [29]. The sow fecal microbiota exerts a persistent influence on the fecal microbiota of offspring piglets until at least 10 days of age [30]. Bacterial and fungal taxa detected in sow feces were also present in piglet gastric and cecal digesta, supporting their role in neonatal gut colonization; *Lactobacillus* largely increased in piglet intestines from days 3 to 7, while post-weaning, plant-glycan fermenters (e.g., *Prevotella-9*) appeared to replace milk–glycan fermenting *Fusobacterium* and *Bacteroides* [31]. Maternal dietary supplementation with *Lactobacillus reuteri* exerted profound effects on the early-life overall microbial composition and maturation of the fecal microbiota in offspring piglets [32]. This included an increased relative abundance of beneficial bacteria in the piglets’ meconium, such as *Romboutsia*, *Lactobacillus*, *Blautia*, *Butyricicoccus*, and *Ruminococcus*. These findings indicate that the initial gut microbiota composition in piglets is primarily governed by the sow, suggesting that modulating the sow’s gut microbiota represents a feasible pathway for indirectly regulating the piglet microbiome.

Analysis of gut microbiota from 613 healthy individuals identified three distinct enterotypes, an *Escherichia shigella*-dominant enterotype, a mixed enterotype characterized by *Bacteroides* and *Faecalibacterium*, and a *Prevotella*-dominant enterotype, with age emerging as the primary driver of microbial variation [33]. The core genus in the vaginal secretions and colostrum of sows was *Pseudomonas*, and in piglets at 1 d of age, *Pseudomonas* and *Escherichia shigella* were most abundant [34]. The gut microbiota of sows on day 3 antepartum was characterized by an increased relative abundance of *Escherichia shigella*, *Fusobacterium*, and *Bacteroides*, alongside a decreased relative abundance of *Alloprevotella*, *Prevotellaceae UCG 003*, and *Ruminococcus 1* [35]. The infant gut microbiota exhibited high heterogeneity, forming three distinct clusters: a *Bifidobacterium*-enriched cluster, a *Bacteroides*-enriched cluster, and an *Escherichia shigella*-enriched cluster [36]. Healthy large white piglets exhibited a continuous decrease in *Lactobacillus* and *Escherichia shigella* alongside a gradual increase in *Prevotella* [37]. This shift in the relationship between *Escherichia shigella* and *Prevotella* could potentially explain the piglet diarrhea. In this study, the relative abundance of *Escherichia shigella* gradually decreased with increasing age in piglets, while there were progressive increases in *Lactobacillus* and *Prevotella*. This microbial shift may contribute to the enhanced disease resilience observed in Chinese indigenous pig breeds.

Distinct differences in gut microbiota composition and richness were observed between lactating sows and non-pregnant/non-lactating Dahe black pigs. The intestinal microbiota of Mangalica pigs exhibited resilience to dietary intake variations (limit feeding vs. ad libitum), and PERMANOVA confirmed that diet significantly influenced microbial community composition (*p* = 0.012, R^2^ = 0.37182) [38]. Significant differences were indeed observed between lactating sows and non-pregnant/non-lactating Dahe black pigs (PERMANOVA, *p* = 0.001, R^2^ = 0.0878); however, the lower R^2^ value compared to dietary factors suggests that physiological stage may have a limited impact on gut microbiota composition. The relative abundance of *Escherichia shigella* was significantly higher in lactating sows compared to non-pregnant/non-lactating pigs. Conversely, lactating sows exhibited significantly lower relative abundances of *Streptococcus*, *Prevotellaceae UCG 001*, and *Clostridium sensu stricto 1*. Furthermore, fecal samples from lactating sows showed significantly higher relative abundances of *Lachnoclostridium*, *Ruminococcus*, *Ruminococcus torques group*, and *Prevotella* than those from non-pregnant/non-lactating pigs. During late gestation in sows, *Rothia*, *Moraxella*, and *Streptococcus* dominated the oral microbiota, while *Clostridium sensu stricto 1* prevailed in the rectal microbiota, and *Streptococcus* emerged as the most abundant genus in both vaginal and colostrum samples [39]. Compared to gestation and empty phases, the lactation stage demonstrated significantly higher relative abundance of bacterial genera, predominantly belonging to Firmicutes (e.g., Lachnospiraceae, *Ruminococcus*) and Bacteroidetes (e.g., Paraprevotellaceae, *Prevotella*) [40,41]. Cellulose-degrading microorganisms in the porcine intestine include *Bacteroides succinogenes* and *Ruminococcus flavefaciens* [42,43]. In the present study, lactating sows of the Dahe black pig breed exhibited higher dietary fiber intake levels compared to the comparative cohorts, which partially explains the observed higher relative abundance of *Ruminococcus* in lactating sows.

Swine fecal bacterial composition varied across growth stages, with *Bacteroidetes* decreasing as weight increased; differential abundance testing between growers and finishers revealed nearly half of the species as shared OTUs, indicating that community differences are primarily driven by abundance variation [44]. Bacteroidetes and Firmicutes were the predominant phyla in the fecal microbiota of healthy pigs at 14, 36, 48, 60, and 70 days of age [45]. *Prevotella*, *Clostridium*, *Alloprevotella*, and *Ruminococcus*, and the *Rikenellaceae RC9 gut group*, were found in 99% of all fecal samples [46]. Compared to older pigs (>90% Firmicutes, ~2% Proteobacteria), one-month-old pigs exhibited significantly lower Firmicutes (73%) and higher Proteobacteria (16.3%), while Bacteroidetes showed fluctuations (5.1%→0.9%→4.9% at 1/2/6 months) and Actinobacteria replaced Spirochaetes as the fourth dominant phylum post-weaning before declining to fifth by six months [47]. Longitudinal analysis of 18 pigs across the lactation (d 0, 11, 20), nursery (d 27, 33, 41, 50, 61), growing (d 76, 90, 104, 116), and finishing stages (d 130, 146, 159, 174) revealed 19 gut microbiome phyla (dominated by Firmicutes and Bacteroidetes) with alpha diversity increasing overall [48]. Gut microbiota in Dahe black pigs exhibited distinct characteristics across growth stages, including an age-dependent increase in the relative abundance of Firmicutes and a gradual decline in Proteobacteria.

The early intestinal colonizers belonging to *Bacteroides*, *Escherichia shigella*, *Clostridium sensu stricto 1*, and *Fusobacterium*, increasing abundances of *Prevotella*, *Butyricimonas*, and *Christensenellaceae R7 group* as the piglets aged, which indicates the adaptation of the piglets to a cereal-based diet rich in oligosaccharides and starch [49]. The predominant genera in suckling piglet microbiota (*Fusobacterium*, *Lactobacillus*, *Bacteroides*, *Escherichia shigella*, and *Megasphaera*) were replaced post-weaning by *Clostridium sensu stricto 1*, *Roseburia*, *Paraprevotella*, *Clostridium XIVa*, and *Blautia* [50]. During suckling, *Lactobacillus* and *Bacteroides* dominated the gut microbiota, with the latter progressively replaced by *Clostridium sensu stricto 1* as piglets aged; upon weaning, the microbiota became enriched with fiber-degrading bacteria [51]. Post-weaning gut microbiome changes rapidly, characterized by decreased relative abundance of *Escherichia shigella* and increased abundance of *Copromorpha*, *Clostridium*, *Fusicatenibacter saccharivorans*, *Intestinibacter*, *Oliverpabstia intestinalis*, *Phascolarctobacterium*, *Prevotella*, and *Ruminococcus* [52]. The fecal microbiota of Pietrain × (Large White × Landrace) castrated male and female pigs’ feces microbiota evolved strongly from 52 to 99 days of age, with an increased abundance of Streptococcaceae and a decreased abundance of Lactobacillaceae; during the finishing stage, microbiota kept evolving at a slower rate [53]. Similar to previous studies, the relative abundance of *Escherichia shigella* in the feces of Dahe black pigs gradually decreased with age, while the relative abundance of fiber-degrading microorganisms (such as *Prevotella*) increased.

Machine learning was applied to feature selection of porcine gut microbiota. Feature selection using random forest impurity-based importance scores identified a core set of 25 to 35 key microbial genera in the Iberian pig, among which *Acetitomaculum*, *Escherichia shigella*, and *Lachnospiraceae FCS020 group* were consistently identified as the most important features across all scenarios [54]. *Mitsuokella*, *Bilophila*, *Succinivibrio*, *Romboutsia*, and *Desulfovibrio* were identified as the top five genera contributing most significantly to distinguishing between the suckling and weaned piglets. Sixty-day-old large white pig populations consistently split into two enterotypes with either *Prevotella* and *Mitsuokella* (PM enterotype) or *Ruminococcus* and *Treponema* (RT enterotype) as keystone taxa [55]. There were higher abundances of *Roseburia*, *Ruminococcus*, *Coprococcus*, *Dorea*, and *Lachnospira* in weaned piglets compared to prior to weaning [56]. The significant post-weaning shift (3–7 days) in the fecal microbiome of all piglets included increased relative abundance of several *Prevotella* spp. and butyrate-producing species (*Butyricicoccus porcorum*, *Faecalibacterium prausnitzii*, *Megasphaera elsdenii*) immediately following weaning [57]. Members of the *Succinivibrio* demonstrated significant negative correlations with growth and carcass traits [58]. In the present study, *Succinivibrio* was significantly enriched in suckling piglets compared to weaned piglets. It is postulated that the early growth and development of Dahe black pigs are linked to the reduced relative abundance of *Succinivibrio*.

*Lachnospiraceae XPB1014 group*, *Clostridium sensu stricto 1*, *Turicibacter*, *Quinella*, and *p 1088 a5 gut group* were the top five contributing microbes discriminating Dahe black weaned pigs and 50–100 kg pigs. *Lachnospiracea XPB1014 group* is a beneficial bacterium involved in the synthesis of short-chain fatty acids [59]. A negative correlation was observed between the *Lachnospiraceae XPB1014 group* and LPS-binding protein levels, which were positively correlated with intramuscular fat (IMF) content [60,61]. *Christensenellaceae R7 group*, *Ruminococcaceae NK4A214 group*, *Lachnospiraceae XPB1014 group*, *Eubacterium coprostanoligenes group*, *Rikenellaceae RC9 gut group*, *Lachnospiraceae AC2044 group*, and *Prevotellaceae UCG 001* comprised the predominant SCFA-synthesizing bacteria [62]. *Lachnospiraceae XPB1014 group* exhibited marked enrichment at the genus level within the colonic microbiota of Shaziling pigs in response to reduced dietary protein [63]. *Prevotellaceae UCG 001*, *Alistipes*, and *Clostridium sensu stricto 1* were highly positively correlated with pig backfat thickness and intramuscular fatness [64]. This indicates that during the ontogenic progression of Dahe black pigs, microbial consortia involved in fiber degradation and lipid metabolism supersede previously dominant taxa to establish themselves as the emergent functional core.

## 5. Conclusions

In conclusion, the intestinal microbiota of Dahe black pigs exhibits fluctuations during the early stages of development. In contrast, the microbiota of growing pigs and finishing pigs demonstrates relative stability. The initial colonization of the piglet gut microbiome is derived from the sow, resulting in similarity between the microbiota of suckling piglets and their dams. Furthermore, the relative abundance profile of the maternal microbiota during lactation differs from that observed in pigs weighing over 120 kg. Utilizing machine learning approaches, we identified two critical transition points in the developmental trajectory of the Dahe black pig microbiota: the shift from suckling to weaning and the transition from weaning to the 50–100 kg growth phase. Key microbial taxa associated with the first transition period (suckling to weaning) include *Mitsuokella*, *Bilophila*, *Succinivibrio*, *Romboutsia*, and *Desulfovibrio*. Key taxa related to the second transition period (weaning to 50–100 kg) comprise *Lachnospiraceae XPB1014 group*, *Clostridium sensu stricto 1*, *Turicibacter*, *Quinella*, and *p 1088 a5 gut group*. These identified microbial taxa represent potential candidate targets for modulating the developmental timing of growth phases in pigs, offering possibilities for either advancing or delaying specific physiological timepoints. Although this study sought to characterize key microbial shifts across different stages, technical constraints of 16S rRNA sequencing preclude reliable resolution of species-level differences. Nonetheless, the identified genus-level signatures delineate a focused scope for subsequent microbiological validation. Future research should prioritize longitudinal tracking of individual pigs to map microbial successions from birth (day 0) through market age.

## Data Availability

The original contributions presented in this study are included in the article/Supplementary Materials. Further inquiries can be directed to the corresponding authors.

## Supplementary Materials

The following supporting information can be downloaded at https://www.mdpi.com/article/10.3390/microorganisms13092038/s1, Table S1: LEfSe analysis of sows and piglets; Table S2: LEfSe analysis in lactating sows and non-lactating pigs; Table S3: LEfSe analysis in suckling and weaned piglets; Table S4: The performance metrics of SP vs WP; Table S5: Lefse analysis in weaned piglets and live weight (50–100 kg) pigs; Table S6: The performance metrics of WP vs. P50-100.

## Abbreviations

The following abbreviations are used in this manuscript:

OTUs	operational taxonomic units
